# Safety monitoring of the RTS,S/AS01_E_ malaria vaccine: experiences and lessons from routine pharmacovigilance in Ghana, Kenya, and Malawi

**DOI:** 10.1186/s12936-026-05889-x

**Published:** 2026-04-04

**Authors:** Adela Ashie, Martha Mandale, Anderson Ndalama, Delese Darko, Seth Seaneke, Eric K. Boateng, George Sabblah, Abena Asamoa-Amoakohene, Kwame Amponsa-Achiano, Naziru T. Mohammed, Rose Jalang’o, Edwin Nkansah, Yvonne Adu-Boahen, Fred Siyoi, Anthony Toroitich, Christabel Khaemba, Pamela Nambwa, Sujeet Jain, Rafiq N. A. Okine, Alambo K. Mssusa

**Affiliations:** 1 Food and Drugs Authority, Accra, Ghana; 2 Pharmacy and Poisons Board, Nairobi, Kenya; 3 Pharmacy and Medicines Regulatory Authority, Lilongwe, Malawi; 4 Expanded Programme on Immunization, Ghana Health Service, Accra, Ghana; 5 National Vaccines and Immunization Programme, Nairobi, Kenya; 6 Pharmacovigilance Unit, World Health Organization, Geneva, Switzerland; 7 Global Malaria Programme, World Health Organization, Geneva, Switzerland; 8 Vaccine Preventable Diseases Unit, World Health Organization Africa Regional Office, Brazzaville, Congo

**Keywords:** AEFI, AESI, Ghana, Kenya, Malaria vaccine, Malawi, MVIP, Pharmacovigilance, Routine PV, RTS,S, Safety

## Abstract

**Background:**

The pilot implementation of the RTSS/AS01_E_ (RTS,S) malaria vaccine in Ghana, Kenya, and Malawi marked a significant step in the fight against malaria. Given the scale of the rollout, which far exceeded the controlled environment of clinical trials, establishing a robust pharmacovigilance (PV) system to monitor rare but potentially serious adverse events following immunization (AEFIs) was a critical component of the program’s design.

**Methods and results:**

In collaboration with technical partners, national health authorities monitored the safety of the RTS,S/AS01_E_ vaccine through strengthened existing routine pharmacovigilance systems and strategies in the three participating countries. Causality assessment of serious AEFIs by national expert advisory committees did not reveal any new safety concerns associated with the RTS,S/AS01_E_ vaccine or confirm the safety signals observed in the Phase 3 clinical trials. The PV efforts successfully supported the safe rollout of the vaccine, contributing to substantial public health benefits in these endemic regions. Challenges encountered included underreporting of AEFIs, delays in data submission and in investigating serious AEFIs, and poor report quality despite targeted investments to strengthen the systems.

**Conclusions and recommendations:**

The pilot program highlighted PV challenges in resource-limited settings and provided useful insights for future vaccine rollouts. The lessons learned from these challenges emphasize the importance of sustained investment, particularly for AEFI investigations, and the need for innovations to improve health worker reporting. Research is needed to identify effective strategies for increasing reporting. Key recommendations include training and capacity building, resource allocation, surveillance and reporting, raising and maintaining public awareness, and collaboration and information sharing to ensure sustained PV efforts and robust safety surveillance in similar contexts.

**Supplementary Information:**

The online version contains supplementary material available at 10.1186/s12936-026-05889-x.

## Introduction

In 2015, the European Medicines Agency (EMA), after reviewing data from a multicentre Phase 3 clinical trial of the RTS,S/AS01_E_ (RTS,S) malaria vaccine, gave a positive scientific opinion under Article 58 [1]. This is equivalent to approving a human medicine intended exclusively for markets outside the European Union. Medicines eligible for this procedure are used to prevent or treat diseases of significant public health interest [2]. This positive scientific opinion confirms that the vaccine is considered safe for public health use under stringent regulatory standards. However, there were outstanding questions, such as the vaccine’s impact on mortality (including sex-specific mortality), that needed to be addressed before a recommendation for broader use could be made. To address the outstanding questions, the World Health Organization (WHO) recommended that pilot implementations using the 4-dose schedule, with rigorous evaluation, be conducted in children aged five months and older in areas with moderate-to-high malaria transmission to assess safety, feasibility, and impact in routine use [3].

Following a call for expressions of interest, Ghana, Kenya, and Malawi were selected to participate in the WHO-coordinated Malaria Vaccine Implementation Programme (MVIP) [4]. The MVIP had two main components: vaccine implementation by the MoH, and the evaluation of vaccine implementation, comprising the WHO-led Malaria Vaccine Pilot Evaluation (MVPE), the GlaxoSmithKline (GSK)-led baseline and Phase 4 studies, and AEFI surveillance through routine PV. The pilot programme was designed to assess the feasibility of implementing four doses of the vaccine in childhood immunization programmes; the vaccine’s safety profile; and the impact of vaccine introduction on mortality and hospital admissions with severe malaria [5, 6]. Data from the pilot informed policymaking at the global and national levels, enabling decisions on the large-scale adoption of RTS,S/AS01_E_ [3].

At the June 2017 meeting, the Global Advisory Committee on Vaccine Safety (GACVS) endorsed six key indicators of vaccine pharmacovigilance readiness that implementing countries agreed to have in place six months before vaccine administration. These indicators included a minimum of 10 AEFI reports per 100,000 surviving infants, a functioning AEFI committee that meets regularly, trained and adequately resourced AEFI investigation teams, evaluated and tested safety communication plans, a designated person within the Expanded Programme on Immunization (EPI) to oversee optimal reporting and training, and established methods for active surveillance of adverse events of special interest (AESIs), with data collection already initiated [7].

The National Regulatory Authorities (NRAs) in Ghana, Kenya, and Malawi authorized the use of the RTS,S/AS01_E_ vaccine for pilot implementation in 2018 through a joint review process facilitated by the WHO African Vaccine Regulatory Forum (AVAREF) [8]. The national EPI-led vaccine implementation started in selected areas in 2019 (April in Ghana and Malawi, and September in Kenya).

The NRAs anticipated that the pilot programme would enable countries to evaluate and strengthen routine vaccine pharmacovigilance. This was expected to generate safety information to inform decision-making about vaccine use and to maintain public trust by providing data to counter fear and misinformation [3]. This paper describes the approach to strengthening routine PV and explores the lessons learnt, gaps, and challenges from routine pharmacovigilance during the pilot implementation of the RTS,S/AS01_E_ malaria vaccine in Ghana, Kenya, and Malawi. The insights will be valuable for new countries planning to strengthen their routine PV systems.

### Safety data collection methods

Health facilities in the implementing regions provided RTS,S/AS01_E_ malaria vaccine as part of routine immunization services to infants and children. The safety of RTS,S/AS01_E_ was monitored through a strengthened routine pharmacovigilance system operating across all of the MVIP areas. The rationale for pharmacovigilance during the MVIP was to identify new safety information arising from the “routine” use of RTS,S/AS01_E_. Routine PV was best placed to detect rare and serious adverse events after vaccination. Such events are too infrequent to be captured or accurately quantified during product development. Two complementary approaches to evaluating safety were also included in the MVIP. Sentinel hospital and community mortality surveillance were established as part of the MVIP to evaluate the safety signals observed during the Phase 3 clinical trials (cerebral malaria, meningitis, and sex-specific mortality [6]. The GSK Phase 4 study was designed to detect adverse events (including meningitis, AESIs, deaths, and other AEs leading to hospitalisation or death) and malaria (including cerebral malaria). A companion paper describes how data from the different surveillance systems were used to inform decision-making [9].

### Regulatory approval of RTS,S/AS01E malaria vaccine

The Food and Drugs Authority (FDA), Pharmacy and Poisons Board (PPB), and Pharmacy, Medicines, and Poisons Authority (PMPA) authorized the malaria vaccine for use in Ghana, Kenya, and Malawi, respectively. These approvals were contingent on strict adherence to national and international safety monitoring guidelines.

### MVIP data safety monitoring board (DSMB)

A programme-specific Data Safety Monitoring Board (DSMB) was set up to protect the well-being of children participating in the MVIP. Quarterly, the DSMB reviewed relevant safety data from the sentinel hospitals, the GSK-sponsored Phase 4 studies, and routine pharmacovigilance across the three countries and provided advice and recommendations to the WHO. Each country appointed NRA focal persons to participate in the DSMB meetings to support the consideration of data from the routine PV systems and to ensure NRA representatives had an overview of the safety of RTS,S/AS01_E_.

### Overview of pharmacovigilance systems in Ghana, Kenya, and Malawi

#### Ghana

Ghana’s National Pharmacovigilance Centre (NPC) is within the Food and Drugs Authority (FDA) [10] The FDA maintains the national safety database, SafetyWatch [11], which is the repository for adverse reaction reports [12]. Vaccine safety reports from health facilities, both private and public, and those received directly from the general population, are all stored in the SafetyWatch system. Reporting of ADRs and AEFIs is mandatory for the marketing authorization holders and voluntary for patients and healthcare professionals. Individual case safety reports (ADRs or AEFIs) received are routinely transmitted to VigiFlow, the WHO safety database. The database is periodically mined for safety signals.

Almost all healthcare facilities have Institutional Contact Persons (ICPs). ICPs are staff members nominated by their management to coordinate pharmacovigilance activities in their institutions on behalf of the FDA and transmit individual case safety reports to the FDA. The FDA has made various reporting tools available. These include paper forms specific to ADRs and AEFIs, as well as electronic reporting tools. The electronic reporting tools include the SafetyWatch System, which is web-based, and the Med Safety App, which is free to use [14]. The FDA also encourages telephone reporting. All these tools are available in versions for both healthcare professionals and patients.

Safety monitoring of AEFIs in Ghana is a collaborative effort between the FDA and the Ghana Health Service (GHS), represented by the EPI. Most spontaneous AEFI reports are submitted to the FDA through the GHS reporting pathway (Figure S1 in Annex), with a few submitted directly by hospitals through healthcare professionals or ICPs.

During the MVIP, the routine system was strengthened through training, job aids, and improved communication to facilitate more effective identification and investigation of AEFIs and to expedite the transmission of reports to the FDA head office. AEFI reports from the district level were expected to be reported simultaneously to the Regional Health Directorate and the Regional FDA Office. Also, GSK and evaluation partners reported AEFIs to the FDA from the GSK-sponsored Phase 4 studies and the pilot surveillance systems, respectively.

A specialised technical advisory committee, the Joint Malaria Vaccine Safety Committee (JMVC), was established by the FDA to conduct causality assessments and safety reviews of all safety data from the three MVIP data sources. All safety reports received were shared with the DSMB and submitted to VigiBase in accordance with the WHO Programme for International Drug Monitoring (PIDM) reporting requirements [13].

Monitoring Adverse Events of Special Interest (AESI) was an essential component of the MVIP, and all health worker training sessions included a dedicated module on this. The same reporting pathway was used for AEFIs (see Figure S1 in the Annex). A list of eleven specific events (see Annex Table S1) was preselected for active monitoring and case detection. The AEFI focal points in the districts of the implementing and non-implementing pilot regions were to conduct surveillance for AESIs.

As part of the AESI surveillance, specialised tools, including AESI reporting forms, case definitions detailing the signs and symptoms of key conditions, and line list templates, were provided to the AEFI regional focal persons. The active case search procedure involved weekly screening of hospital records to identify children who met the case definitions for the preselected AESIs. Additionally, regional EPI Coordinators, FDA officers, and national-level EPI and FDA staff routinely reviewed all AEFI reports as an additional layer of screening to initiate the necessary investigation. Clinicians were also identified to support follow-up and patient management, ensuring that complete clinical records and investigation reports were available for thorough causality assessment.

#### Kenya

In Kenya, through the National Pharmacovigilance Centre (NPC), the Pharmacy and Poisons Board collects, collates, analyses, and communicates safety information on health products and technologies authorized in Kenya.

This national reporting system includes paper-based and electronic reporting tools, a national database, and two safety expert Committees: the Pharmacovigilance Expert Review and Advisory Committee and the National Vaccine Safety Advisory Committee.

The Pharmacovigilance electronic reporting system (PvERS) [15], compliant with ICH E2B standards, is used to manage individual case safety reports from the public, healthcare professionals, Marketing Authorization Holders (MAHs), sentinel sites, and Public Health Programs. The mobile PvERS (mPvERS) application, available on Android and iOS, also facilitates adverse event reporting. To enhance consumer reporting, the NPC has a dedicated telephone line and Unstructured Supplementary Service Data (USSD) code for reporting adverse events. As a WHO PIDM member, the NPC shares safety information through VigiBase. Data from reports following vaccination with the RTS,S/AS01E (RTS,S) malaria vaccine were also shared with the DSMB and the WHO.

The NPC collaborates with the Ministry of Health, Public Health Programs, sentinel sites, County governments, the pharmaceutical industry, Development Partners, and the WHO.

The routine Pharmacovigilance (PV) system was utilised to monitor and report AESIs related to the malaria vaccine, following standard approaches to reporting AEFIs. This system relied on healthcare professionals in the targeted regions to identify, document, and report any AESIs occurring after vaccination. To facilitate this, healthcare professionals were trained to detect and report AESIs. The training, which covered topics including vaccine administration, cold chain management, community engagement, and monitoring and reporting of adverse events following immunization, was delivered to healthcare professionals at both managerial and service-delivery levels. The training specifically targeted healthcare professionals in the eight selected MVIP Counties.

The National Vaccine Safety Advisory Committee (NVSAC) evaluated the causality of reported AEFIs/AESIs and provided expert recommendations on risk management strategies. The AEFI reporting system of the PPB, which was the same approach used during the MVIP, is presented in Figure S2 in the Annex.

#### Malawi

In Malawi, the NPC serves as the secretariat for all pharmacovigilance activities through the Pharmacy and Medicines Regulatory Authority (PMRA). Patients report any adverse events to the nearest health facility or healthcare worker. Alternatively, patients can report directly to the PMRA via the Medsaf-360 USSD platform [16] or the patient reporting form. MAHs, disease control programs, and pharmaceutical outlets (hospitals, pharmacies, and medicine stores) detect, investigate, manage, and report to the PMRA all safety cases, including adverse events following immunization and suspected adverse drug reactions. The Medicines Safety and Quality Monitoring Committee (MSQMC) reviews and analyzes reports of adverse events and performs causality assessments of adverse events presented to the committee by the NPC. Each hospital has a designated pharmacovigilance coordinator and pharmacovigilance focal point. Individual Case Safety Reports (ICSRs) are reported through these focal points to the PMRA NPC. Safety case reports are submitted to the NPC using standard reporting forms [17].

The PMRA’s AEFI reporting system was used during the MVIP and is presented in Figure S3 (Annex). Sources of AEFI reports were similar across the three implementing countries. As part of the protocol, data collected during the MVIP were shared with the DSMB and the WHO, and thus with the NRAs of the three participating countries, whose representatives attended the DSMB meetings.

### Key stakeholders and collaborators, and their roles

The implementation of safety surveillance involved several key stakeholders and collaborators across the participating countries. In Ghana, stakeholders included the Ghana Health Service (GHS), the EPI, the WHO, the FDA, and several research institutions, including the Kintampo and Navrongo Health Research Centres and the Noguchi Memorial Institute for Medical Research (Table 1).

**Table 1:** Stakeholders and their roles in the MVIP Safety Monitoring

Category of stakeholder	Role	Ghana	Kenya	Malawi
National stakeholders	Implementation and safety monitoring of the vaccines. Review of safety data for decision making	Food and Drugs Authority Ministry of Health Ghana Health Service Expanded Programme on Immunization National Malaria Control Programme	Pharmacy and Poisons Board National Vaccines and Immunization Programme	Pharmacy and Medicines Regulatory Authority Ministry of Health Expanded Programme on Immunization
Funding Agency	Provision of funds for vaccine deployment and safety monitoring activities Provision of technical support: training and guidelines	WHO PATH	WHO PATH	WHO PATH UNICEF Malawi Liverpool Wellcome Trust (MLW)
Researchers	Conduct of Phase 4 safety monitoring Reporting of safety data to the NRA from sentinel hospitals as part of routine PV reporting for review and decision making	Kintampo Health Research Centre Navrongo Health Research Centres, Noguchi Memorial Institute of Medical Research	Kenya Medical Research Institute	Kamuzu University of Health Sciences

The National Vaccines and Immunization Programme (NVIP), the PPB, healthcare professionals from the selected facilities, the WHO country office, PATH, and the Kenya Medical Research Institute (KEMRI) were central to the programme’s implementation in Kenya.

In Malawi, the National Pharmacovigilance System comprises the Ministry of Health, NPC at PMRA, MSQMC, Public Health and Disease Control Programs under the Ministry of Health, Hospitals, clinics, pharmacies and medicine stores, MAH, and the public (patients and caregivers). The EPI, PMRA, the MOH, PATH, WHO, UNICEF, Malawi Liverpool Wellcome Trust (MLW), the National Malaria Control Program, Kamuzu University of Health Sciences (KUHES), and various healthcare professionals and media partners supported implementation efforts.

### Safety data collection during MVIP

In Ghana, electronic databases, the MedSafety App, and online reporting systems were utilized alongside traditional paper forms, allowing efficient data entry and retrieval.

Kenya and Malawi leveraged similar tools, with electronic reporting systems capable of managing potentially large volumes of data. These systems were designed to integrate seamlessly with existing healthcare infrastructure, ensuring that AEFI data was easily accessible for analysis and decision-making.

### AEFI investigation and causality assessment

Case investigations were conducted for serious AEFIs, clusters, significant events of unexplained cause, increases in the number or rates of known adverse reactions, or events following immunization that caused significant parental or public concern. In all three countries, an AEFI investigation team was formed, which included a representative from the NRA, EPI, and the district director of public health or a public health nurse.

In Kenya, representatives of the WHO and the United Nations International Children’s Emergency Fund (UNICEF) joined the investigation teams as needed to provide technical support. In Ghana, the AEFI investigation team was established at the regional level and included AEFI focal persons from the district where the investigation was being conducted. However, in Kenya and Malawi, the AEFI investigation team was at the district level. An AEFI investigation is expected to be initiated within 24–48 h of the team’s notification, and the preliminary report is expected to be ready within 7 days. The WHO AEFI Aide-memoire guided the investigation on AEFI.

The process involved multiple steps, which included reviewing medical records, interviewing of vaccinators, healthcare providers and guardians, observing the vaccine management practices, and collecting the necessary samples, including vaccine vials-both open and unused (closed), injection devices, diluents, relevant clinical samples-blood, swabs, biopsies, and other clinical investigations, and conducting site visits to immunization clinics where the vaccination took place. The information collected was recorded on the WHO AEFI investigation form.

**Fig. 1:**
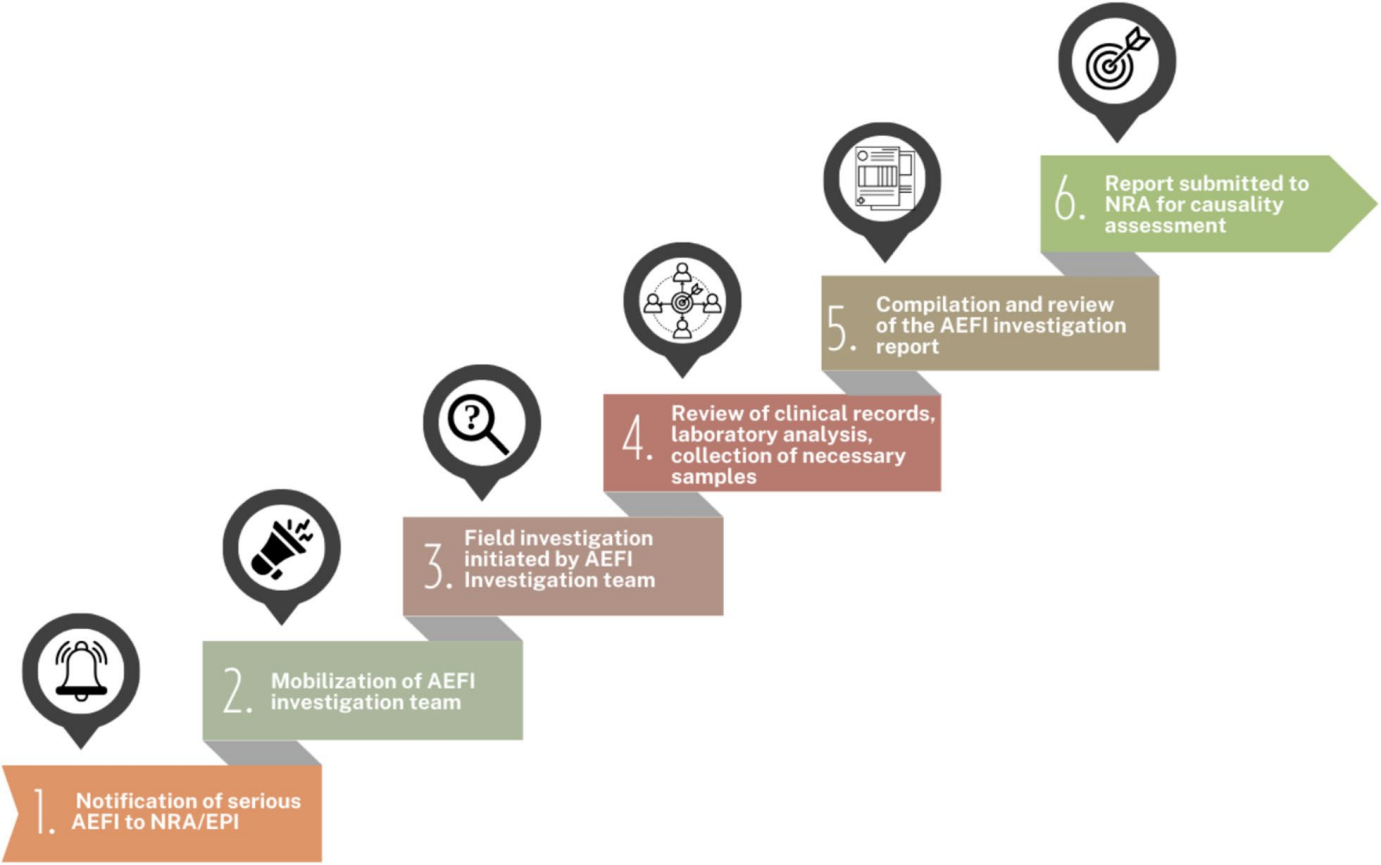
Process for investigating serious AEFI, from notification to causality assessment

The completed AEFI investigation forms, narrative report, copies of hospital case notes and records, laboratory findings, and autopsy reports were sent to the NRA. The NRA reviewed and prepared the investigation report for presentation to the expert committees for causality assessment and, as necessary, recommendations. The DSMB members were then briefed on the outcomes of the causality assessment conducted by the in-country expert committee (Fig. 1).

The investigation process for serious AEFI began with the NRA and EPI notification, which immediately informed the AEFI investigation team. Every effort was made to initiate the investigation within 24–48 h and conduct a visit to assess the event. Findings were then submitted to the NRA for causality assessment, after which feedback was provided to the original reporter. If the report was received via that system, it was processed through the EPI.

Causality assessments were conducted for serious AEFIs to determine whether the vaccine was causally associated with the AEFIs. The causality assessment involved a detailed review of the clinical data, the temporal relationship between vaccination and the adverse event, and any other relevant information. The JMVC, NVSAC, and MSQMC in Ghana, Kenya, and Malawi, respectively, conducted the causality assessment. These committees comprise experts in epidemiology, pharmacovigilance, paediatrics, and infectious diseases. The committees used the electronic WHO causality assessment tool. [18] for causality assessment.

**Fig. 2:**
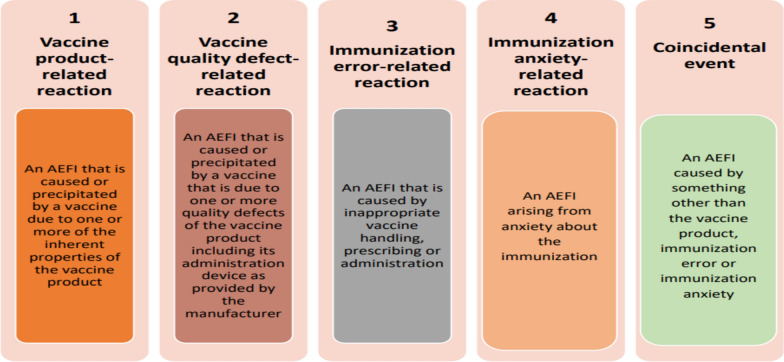
Cause-specific definitions of AEFIs. Source: WHO

The causality assessment outcomes of the AEFI reports were systematically documented and categorized as vaccine product-related reactions, vaccine quality defect-related reactions, immunization error-related reactions, immunization anxiety-related reactions, or coincidental events (Fig. 2).

### Safety data received at NRAs

The total number of AEFIs reported over the period (2019–2023) was 2292, 1623, and 329 in Ghana, Kenya, and Malawi, respectively (Table 2). In Ghana, 96% of the reports were received from EPI MAL-003 (GSK Phase 4), and 2% each from MVPE sentinel hospitals and National PV. In Kenya, 68% of the reports were received from MVPE sentinel hospitals, 30% from EPI MAL-003, and 2% from National PV. In Malawi, 54% of the reports were received from EPI MAL-003 and 46% from National PV. No reports were obtained from the MVPE arm in Malawi (Table 3).

**Table 2:** Doses of RTS,S/AS01_E_ administered and AEFIs reported during the pilot (2019–2023)

Country	Total doses administered	Total AEFIs reported	AEFI per 100,000 doses
Ghana	2,569,752	2292	89.2
Kenya	1,818,572	1623	89.3
Malawi	2,193,678	329	15

**Table 3:** RTS,S/AS01_E_ malaria vaccine AEFIs received in Ghana, Kenya, and Malawi regulatory authorities (2019–2023)

	Ghana				Kenya				**Malawi**			
Source	Total AEFIs^N^ (% source)	Serious^n^ (n/N%)	Causality Assessed^a^ (a/n%)	Fatal^b^ (b/n%)	Total AEFIs^N^ (% source)	Serious^n^ (n/N%)	Causality Assessed^a^ (a/n%)	Fatal^b^ (b/n%)	Total AEFIs^N^ (% source)	Serious^n^ (n/N%)	Causality Assessed^a^ (a/n%)	Fatal^b^ (b/n%)
EPI-MAL-003	2196 (95.8%)	411 (18.7%)	354 (86.1%)	23 (5.6%)	491 (30.3%)	394 (80.2%)	42 (10.7%)	109^§^ (27.7%)	178 (54.1%)	178 (100%)	37 (20.8%)	47 (26.4%)
National PV	42 (1.8%)	10 (23.8%)	7 (70%)	1 (10%)	30 (1.8%)	2 (6.7%)	2 (100%)	0 (0%)	151 (45.9%)	6 (4%)	0 (0%)	0 (0%)
MVPE	54 (2.4%)	46 (85.2%)	35 (76.1%)	2 (4.3%	1102 (67.9%)	1102^*^ (100%)	72 (6.5%)	265 (24%)	0 (0%)	0 (0%)	0 (0%)	0 (0%)
Total	2292 (100%)	467 (20.4%)	396 (84.8%)	26 (5.6%)	1623 (100%)	1498 (92.3%)	116 (7.7%)	374 (25.0%)	329 (100%)	184 (55.9%)	37 (20.1%)	47 (25.5%)

*All the reports from the MVPE (hospital surveillance) were categorized as serious because they were from hospitalized patients

^§^The fatal cases were not causally associated with the RTS,S vaccine

The number of AEFIs reported per 100,000 doses was similar in Ghana and Kenya (~ 89 per 100,000 doses) and was comparatively lower in Malawi (15 per 100,000 doses) (Table 2). The detailed breakdown of the AEFIs received from each country is presented in Table 3

**Fig. 3:**
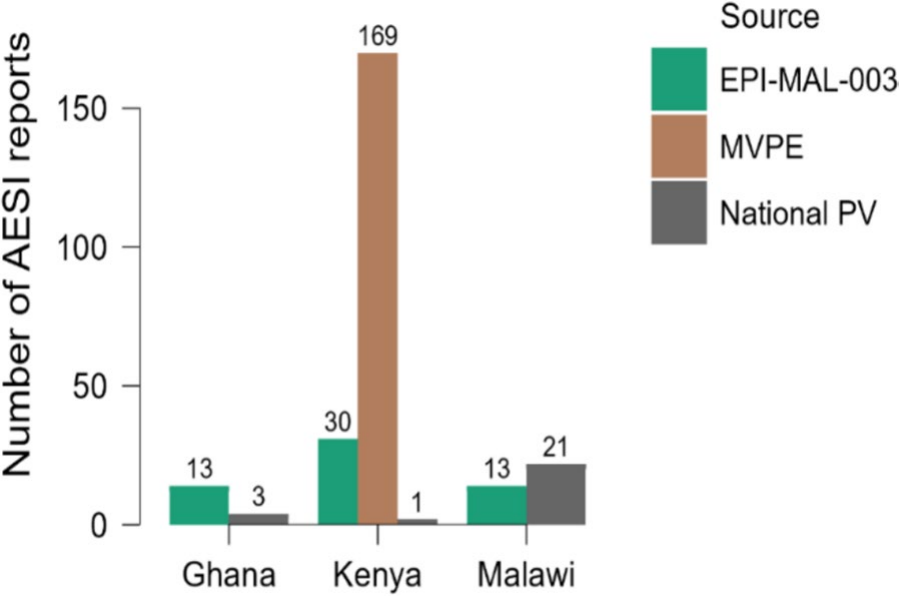
AESI reports, 2019–2013

A total of 250 AESIs were documented, 16 in Ghana, 200 in Kenya, and 34 in Malawi. The distribution of the AESI reports by source and country is presented in Fig. 3.

**Fig. 4:**
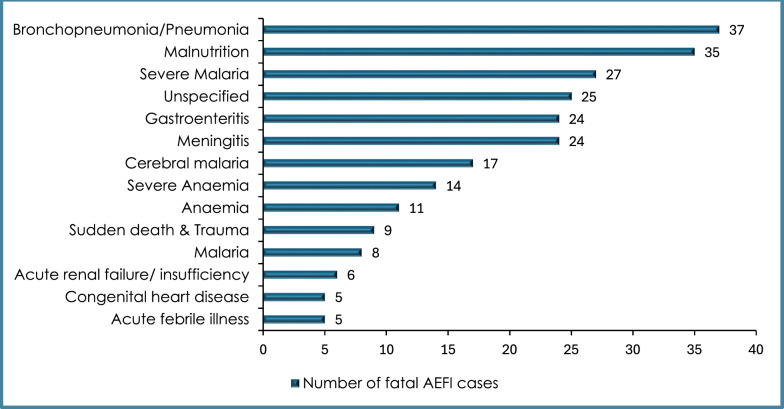
Frequencies of top 15 fatal RTS,S/AS01E malaria vaccine AEFIs received in Ghana, Kenya, and Malawi regulatory authorities (2019–2023) (N = 247)

A total of 529 fatal AEFIs were reported from the three countries between May 2019 and September 2023. The frequencies for the top 15 fatal AEFIs are presented in Fig. 4. These fatal cases were not causally associated with the RTS,S vaccine.

**Fig. 5:**
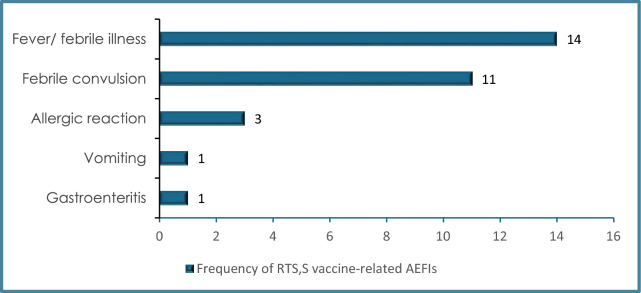
Frequency of reported cases assessed as A1-Vaccine Product Related Reaction received at Ghana and Kenya regulatory authorities (2019–2023). None of the AEFIs in Malawi were evaluated to be vaccine product-related (N = 30)

Of the 4,244 AEFIs reported from the three countries, 2,149 (50.7%) were serious, and 2,095 were non-serious. Of the 2,149 serious AEFIs, 549 (26%) were causally assessed, with rates ranging from 8% in Kenya to 20% in Malawi and 85% in Ghana. Of the 549 causally assessed AEFIs, 30 were deemed to be Vaccine Product Related Reactions (Fig. 5).

### Lessons and challenges during programme implementation

To effectively support the pilot programme, each participating country implemented measures tailored to improve collaboration, coordination, communication, and training among key stakeholders, ensuring robust monitoring and response mechanisms for vaccine safety.

Implementing PV strengthening activities was integral to ensuring effective safety surveillance across the three countries. The NRAs played a crucial role in overseeing PV activities, holding regular supervision meetings to review progress and address emerging challenges. These meetings ensured continuous oversight and provided a platform for stakeholders to discuss and refine their strategies. The majority of the challenges experienced by the countries were similar to those observed in other new vaccine introductions in sub-Saharan Africa, e.g., MenAfriVac and HPV.

### AEFI surveillance

In Ghana, collaboration and communication among the EPI, FDA, researchers, and GSK were enhanced, and AEFI focal points were established. To improve the identification and management of AEFIs, training programmes were organized for healthcare professionals in all participating regions on the AEFI surveillance cycle and their roles and responsibilities. While there was collaboration with the EPI at the national level, which has been in place for 20 years since the introduction of the pentavalent vaccine, challenges persisted at the regional and lower levels. The less effective collaboration at these subnational levels weakened efforts to strengthen the AEFI surveillance system.

In Kenya, PV system strengthening activities included appointing focal persons for the MVIP DSMB meetings, establishing and operationalizing the NVSAC, and monthly MVIP coordination meetings. Capacity-building efforts included training healthcare professionals in AEFI monitoring and reporting, training NVSAC staff in causality assessment, and training counties and sub-counties in investigating serious AEFIs. Additionally, supportive supervision of selected facilities implementing the MVIP was conducted, with a focus on the MVIP. Information, Education, and Communication (IEC) materials were developed by the National Vaccines and Immunization Program and distributed to healthcare providers. Kenya encountered several challenges that affected the efficiency of its AEFI surveillance system. Financial constraints limited the capacity to provide ongoing training for healthcare professionals in AEFI monitoring and reporting. Additionally, there were significant delays in submitting paper-based AEFI reports at the national level, creating gaps in the safety monitoring process and delaying timely responses to safety concerns.

Malawi improved collaboration with national and international partners, including the WHO, EPI, MVPE, and NRAs. PV strengthening activities focused on training healthcare professionals on AEFI surveillance, AEFI investigation teams, and the MSQMC on causality assessment and holding data reconciliation meetings between the PMRA and EPI. The MOH, EPI, and PMRA conducted public awareness campaigns and training sessions on AEFI surveillance. The EPI officers and the PMRA, with support from WHO and PATH, conducted multiple PV training sessions. At least 20 personnel per district, across all 11 implementing districts, were trained in AEFI surveillance.

### AESI surveillance

#### Ghana

AESI surveillance was incorporated into the routine activities of the regional AEFI focal persons to be performed in addition to all other functions. The increased workload, the novelty of AESI surveillance, and challenges with applying the case definitions negatively impacted AESI surveillance. A dedicated team of at least two personnel per region, working solely on AESI identification, might have improved identification and reporting. Additionally, the misconception among districts that they were not deploying the RTS,S/AS01_E_ vaccine and that they were exempt from the AESI case search could have been mitigated by integrating these districts into the regional focal persons’ monthly reporting targets.

Other challenges with the AESI monitoring included low identification rates and delayed reporting of AESI cases. In some instances, cases flagged at the national level based on AEFI forms were not recognized as AESIs at the regional level, which delayed timely investigation. Furthermore, there was limited identification and reporting of AESIs from districts that did not deploy the vaccine, even though they were in regions where the pilot was being implemented. This affected the comparison of AESI incidence rates between vaccinated and unvaccinated children. Regular reminders were sent to the regional focal persons to encourage screening for AESIs and ensure the availability of reporting tools and case definitions. However, these efforts yielded minimal improvements, as the case definitions did not effectively facilitate identification.

#### Malawi

A functional AESI surveillance system could not be established in accordance with the AESI surveillance guidelines. As a result, AESI surveillance was conducted in the same manner as AEFI surveillance. The key challenge in the absence of a separate AESI surveillance system was a lack of financial resources, particularly in the early years. Although the African Field Epidemiology Network (AFENET) received CDC support for some system-strengthening activities, available funds were insufficient to sustain all of them regularly.

#### Kenya

Underreporting was a significant issue, as seen in other vaccine introductions, and was common, especially in passive surveillance systems. This was exacerbated by healthcare professionals’ competing responsibilities, time constraints, and varying levels of knowledge about AESI detection and reporting.

Although training programs were implemented to address these gaps, limited resources and funding significantly hindered their effectiveness. Many healthcare professionals require additional training to recognize and report AESIs accurately. Follow-up on reported cases was often inconsistent due to logistical challenges, including personnel shortages and insufficient funding.

### AEFI data collection and case reporting

In Ghana, stakeholder engagements were held with GSK Phase 4 study investigators, MVPE partners, and the EPI to ensure all stakeholders understood the mandatory reporting requirements for the Phase 4 study investigators. The aim was to strengthen data collection and sharing. Insufficient training and low awareness among clinicians regarding the AEFI reporting system were significant challenges in Ghana, as previously observed in other vaccine introductions [19] which led to underreporting of AEFIs. Furthermore, delays in submitting AEFI reports through the healthcare system created bottlenecks, further hindering the timely analysis and response. The MVPE was based on the routine reporting system and could account for the similar AEFI reporting rate observed in the routine system.

In Kenya, underreporting of AEFI cases was also common [20]. The low reporting from routine PV in Kenya was due to the passive, voluntary nature of the reporting, which often leads to underreporting. Other reasons included the perception that only serious or unusual events warrant reporting [21]; high workloads; competing tasks among healthcare professionals; and caregiver health-seeking behaviour, leading to underreporting of many community events. Additionally, time constraints from competing tasks and limited human capital further contributed to this underreporting [22]. Delays in submitting reports from healthcare facilities to the national level were similar to those observed in Ghana. [23]. Moreover, budgetary constraints limit the ability to continuously sensitize healthcare providers to the monitoring and reporting of AEFIs.

In Malawi, the challenges in AEFI data collection and reporting were similar to those in Ghana. Insufficient training and low awareness of the reporting system among clinicians led to significant underreporting of AEFIs. Delays in submitting reports to the next level further compounded the issue, creating inefficiencies in the pharmacovigilance process and delaying necessary actions to address potential safety concerns. The MVPE investigators did not provide reports because AEFI reporting was not included in the study protocol.

Overall, the implementation program highlighted the perennial issue of underreporting of AEFIs observed in other vaccine introductions [11, 24–26] which NRAs have been attempting to address through various strategies over the years.

### AEFI data management

Common challenges across all countries included delays in submitting reports to the next level of the healthcare system and partially completed reports, both of which hindered timely data analysis and causality assessment [27, 28].

Additionally, in Ghana, insufficient details in clinical records, especially from Phase 4 investigation sites, posed another significant challenge. The investigations often did not utilise the WHO AEFI investigation report or aide-mémoire, resulting in limited data quality and comprehensiveness. The poor quality and completeness of the reports affected the outcome of the causality assessment [29]. Continuous engagement and feedback were employed to help address the issue. The investigators were required to promptly notify the FDA to ensure the Regional Investigation Team was involved in the serious AEFI investigation and that all the necessary data were collected.

Reporting was generally paper-based because the health system structure required the form to pass through multiple administrative levels. However, in Ghana, for example, an electronic version may be submitted directly to the national level via email or WhatsApp.

### AEFI investigation

Specific training on AEFI investigation was organised for regional AEFI investigation teams to improve the quality of the investigations. In Ghana and Kenya, delays in investigating serious AEFI cases occurred due to late notification and insufficient resources for comprehensive investigations [29]. In most cases, though, it took the team a few days to be mobilised to initiate the exercise. This delay was due to staff being engaged in competing activities or to limited resources for the investigation.

The WHO provided funding that partially improved field investigations in Ghana; however, the delay in notifying serious AEFIs affected the quality of the findings. This hindered the ability to respond promptly and thoroughly to serious adverse events. In Malawi, seven personnel per district were trained in AEFI investigation to form the district AEFI investigation team. Delays in reporting and resource constraints compounded the challenges in the AEFI investigation. Investigation of serious cases was delayed because of the timing (monthly) of the case line list submission.

### AEFI causality assessment

The members of the advisory committees who conducted causality assessments for the safety reports in each country were trained to ensure they fully understood the MVIP, its objectives, and rationale, enabling them to provide informed and practical guidance.

In Ghana, delays in investigating serious AEFI cases significantly affected the timeliness and outcomes of causality assessments, hindering the prompt identification and resolution of potential vaccine-related issues. Additionally, resource constraints affected the overall conduct of investigations, further complicating the timely assessment of causality [30]. Inadequate clinical information, lack of comprehensive lab investigations, and insufficient AEFI investigations at lower levels of the healthcare system [27, 28, 31] contributed to the sub-optimal quality of the causality assessments.

Kenya faced similar issues due to insufficient information on serious AEFI cases. Accurate causality assessment relies heavily on detailed, comprehensive data [32]; however, the lack of such data poses a significant barrier. Without complete and reliable clinical data, lab results, and thorough investigations, making informed decisions regarding the causality of reported AEFI cases was challenging. The lower rates of causality assessment of serious AEFIs in the EPI MAL-003 in Kenya were because AEFI data in the EPI MAL-003 arm were assessed and reported by the principal investigators to the PPB. This process was outside of routine AEFI workflows. Causality assessment by the safety committee (NVSAC) required that serious AEFI cases be investigated by county investigation teams using the WHO-standardized AEFI investigation form.

In Malawi, the major challenge for causality assessment was that the MSQMC was unable to discharge its duties for an extended period due to funding shortages and technical gaps, resulting in a backlog of work. Technical support was later obtained.

### AEFI communication

Advocacy and social mobilization were key to the MVIP’s public education strategy. Audio clips for social media discussions were created to raise public awareness about the vaccine. The Ghana Health Service used its social media platforms to disseminate information to parents and caregivers. Specific education materials were also created for healthcare professionals, including vaccinators. In addition, spokespersons from key institutions, including the MoH, EPI, and FDA, were trained to ensure consistent and accurate messaging. These spokespersons also participated in media engagements on radio and TV morning shows to educate the public and address questions. Additionally, FAQs were developed and published on the FDA’s website and circulated on the FDA’s social media platforms to provide information on the malaria vaccine, safety, and the MVIP. A crisis communication plan was also developed to guide the vaccine safety communication before, during, and after the MVIP. This helped ensure a prompt response to issues that could escalate into crises.

In Ghana and Malawi, one of the primary challenges was ensuring timely feedback on causality assessments for stakeholders, including reporters and the EPI, which hindered prompt action and follow-up. Furthermore, misinformation and disinformation about the vaccine’s safety spread across various platforms, particularly social media, complicating efforts to provide accurate information and manage public perception. Although many pre-vaccination advocacy, communication, and social mobilization activities were implemented, it was challenging to address the social media rumours. Limited resources to purchase airtime on media further constrained the dissemination of essential information to the public.

Kenya also faced considerable challenges, particularly in addressing misconceptions about the implementation program. Inadequate resources hindered the feasibility of conducting continuous awareness campaigns to educate and inform the public.

### Strategies employed by countries to overcome challenges

To address underreporting and delays associated with paper-based reporting forms in Ghana, healthcare workers were trained during the FDA’s routine sensitization programs, which focused on identifying AEFIs and the importance of timely reporting. Hands-on sessions on completing an AEFI using a dummy case were included in AEFI training to address issues with the poor quality and completeness of reports received. There was a move to conduct more training in lower-level healthcare facilities and to avoid relying solely on cascaded training. This resulted in improved quality and increased the number of reports received. This reduced information dilution as it moved from the higher level to the facility level. Additionally, issues with the quality of AEFI investigations were addressed during FDA supervision visits to MVIP sites. A positive response from the WHO to fund the training of AEFI investigation teams and the conduct of field investigations helped mitigate these challenges. WHO additionally provided funding for the JMVC meetings. The combination of these interventions improved the timeliness of communicating causality assessment outcomes to reporters.

In Kenya, the PPB integrated the AEFI reporting form into the PvERS with seed funding from the WHO. Additionally, with support from the USAID Medicines, Technologies, and Pharmaceutical Services (MTaPS) Program, the PPB developed a USSD code to enhance consumer reporting. A dedicated toll-free telephone line is a good starting point for consumer reporting. The PPB also incorporated a vaccine safety surveillance module into the PV training curriculum, and healthcare professionals were trained on this module during routine PV sessions to supplement cascaded training. Furthermore, the WHO facilitated NVSAC meetings to conduct causality assessments of serious cases that had been investigated.

In Malawi, stakeholder meetings helped address most of the implementation challenges. Through this engagement, the issue of data reconciliation with GSK was addressed, and case investigations and causality assessments were conducted with support from the WHO country office. In contrast, healthcare worker training, AESI case investigation, adjudication, supervision, and mentorship were conducted with support from the AFENET.

To address data management challenges across all three countries, the NRAs, the MVPE partners, and the GSK phase 4 investigators coordinated effectively to ensure that reports from these other sources were transmitted to the NRAs.

An initiative that sustained the momentum of safety monitoring activities was the participation of NRA representatives in MVIP-specific DSMB meetings. This ensured that countries continuously monitored the three safety data sources (MVPE, phase 4 studies, and routine PV). These meetings, which GSK Phase 4 researchers also attended, provided a platform for clarifying procedural variations across countries. This provided the basis for the NRAs not requesting safety data from the researchers, having observed that this was being done in other countries. Triangulating data from all three sources and sharing information between the NRAs improved the understanding of the safety data.

## Recommendations

### The recommendations discussed below address the challenges faced by all three countries.

#### Training and capacity building

Training efforts should focus on AEFI investigation teams to ensure they are well-equipped to gather and report high-quality information. Clinicians require specific training to improve their identification of a case as an AEFI, to emphasize the need for complete clinical investigations and detailed record-keeping for suspected AEFI cases [31, 33]. Additionally, DSMB members should receive training on vaccine safety monitoring in the post-marketing phase, regulatory requirements, and the implementation context. Capacity building should also include enhancing public and healthcare professionals’ awareness of AEFI reporting channels and the importance of reporting.

#### Resource allocation

Balanced resource allocation is critical to support safety surveillance activities. Currently, resources are often skewed towards logistical and public relations activities during mass vaccination campaigns [34, 35]. A more balanced approach is needed to ensure sufficient funding for training, AEFI investigation, causality assessment, and other pharmacovigilance activities to strengthen the underlying PV systems [33]. This includes providing adequate resources for timely, comprehensive investigations of serious AEFI cases and facilitating ongoing education for healthcare professionals.

#### Surveillance and reporting

Strengthening active surveillance systems to detect rare or delayed AEFIs is vital. Promoting the use of digital tools [36], for example, the MedSafety application, can streamline and enhance AEFI reporting efficiency. Efforts should also focus on addressing underreporting by educating healthcare professionals on the importance of reporting all AEFIs, including non-serious events [25, 31] and overcoming time constraints due to competing tasks. Ensuring the timely submission of AEFI reports from facilities to the national level and improving their completeness and quality are essential steps that can be achieved through continuous education [35, 37].

#### Public awareness and communication

Continuous awareness campaigns should be conducted to inform the public and healthcare professionals about vaccine safety and the importance of monitoring and reporting AEFIs [38]. Public awareness campaigns should be conducted during vaccination campaigns and even when no campaign is scheduled to prevent the aims of these campaigns from being misinterpreted. Addressing misinformation and misconceptions about vaccines through targeted communication strategies will help build public trust in vaccination programs [39].

#### Collaboration and information sharing

Strengthening collaborations and safety information sharing among relevant stakeholders is necessary to enhance pharmacovigilance systems [36, 37]. This includes fostering in-country collaborations and ensuring effective communication and feedback mechanisms between reporters, regulatory authorities, and other stakeholders [36, 40].

## Conclusion

Implementing the RTS,S/AS01_E_ malaria vaccine pilot program in Ghana, Kenya, and Malawi has provided valuable insights into the capabilities and challenges of routine pharmacovigilance. Strengthened pharmacovigilance systems and capacity-building initiatives have significantly enhanced the monitoring and reporting of AEFIs, thereby contributing to a more robust and responsive vaccine safety surveillance system. Despite challenges, including delays in reporting, underreporting of AEFIs, and resource constraints, the pilot program has strengthened the foundations for future vaccine safety monitoring. It has provided critical lessons for the introduction of the RTS,S/AS01_E_ vaccine. The experiences gained from this pilot may help other countries planning to strengthen PV activities to support the introduction of new vaccines and other health interventions.

## Supplementary Information

Additional file1

## Data Availability

Data will be made available on request.

## Abbreviations

AEFI: Adverse events following immunization
AESI: Adverse events of special interest
AFENET: African field epidemiology network
DSMB: Data and safety monitoring board
EMA: European Medicines Agency
EPI: Expanded programme on immunization
FDA: Food and drugs authority
GACVS: Global advisory committee on vaccine safety
GHS: Ghana health service
GSK: GlaxoSmithKline
ICSRs: Individual case safety reports
ICH: International Conference on Harmonisation
KEMRI: Kenya Medical Research Institute
KUHES: Kamuzu University of Health Sciences
MAH: Marketing authorisation holder
MLW: Malawi Liverpool Welcome Trust
MOH: Ministry of Health
MPAG: Malaria Policy Advisory Group
MVIP: Malaria Vaccine Implementation Program
MVPE: Malaria Vaccine Pilot Evaluation
MSQMC: Medicine Safety and Quality Monitoring Committee
NPC: National Pharmacovigilance Center
NRA: National Regulatory Authority
NVIP: National Vaccines and Immunization Programme (NVIP)
NVSAC: National Vaccines Safety and Advisory Committee
PIDM: WHO Programme for International Drug Monitoring
PMRA: Pharmacy and medicines regulatory authority
PMPB: Pharmacy, medicines and poisons board
PPB: Pharmacy and poisons board
PV: Pharmacovigilance
SAGE: Strategic advisory group of experts
SSA: Sub-Saharan Africa
TAC: Technical Advisory Committee
UNICEF: United Nations International Children’s Emergency Fund
USSD: Unstructured supplementary service data
WHO: World Health Organization
