# Supplementary material for: Safety monitoring of the RTS,S/AS01E malaria vaccine: experiences and lessons from routine pharmacovigilance in Ghana, Kenya, and Malawi
Source: Malar J. 2026 Apr 4;25:197. doi: 10.1186/s12936-026-05889-x (PMC13147739; doi:10.1186/s12936-026-05889-x)
Supplement: Supplementary file 1 — Additional file1 [file 12936_2026_5889_MOESM1_ESM.docx]

**Annexes**

Table S1: List of AESIs monitored by countries during the MVIP


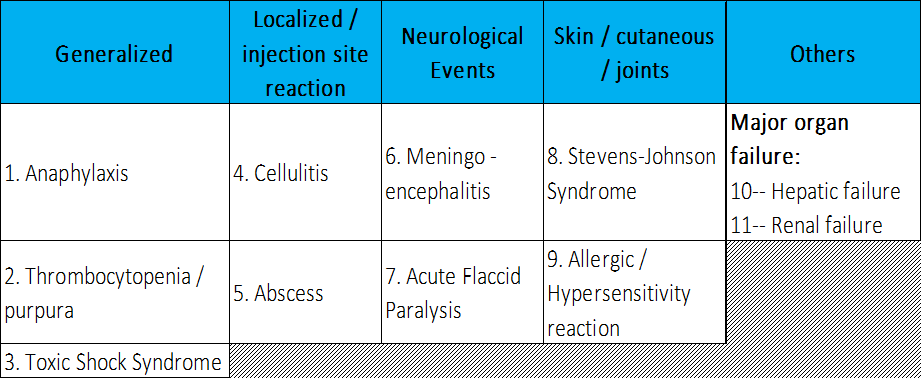


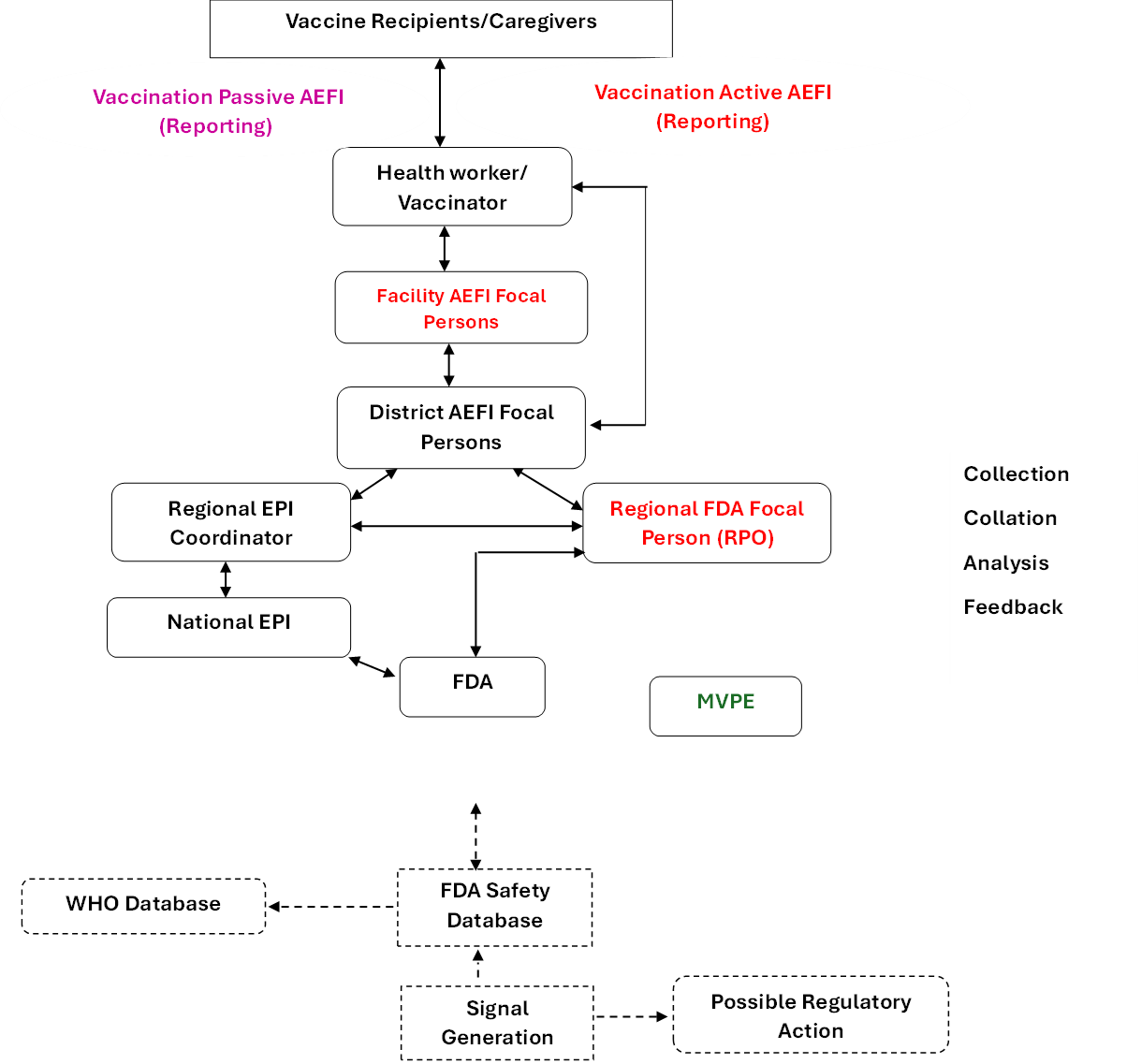


**JMVC**

**GSK**

Figure S1: AEFI reporting organogram in Ghana


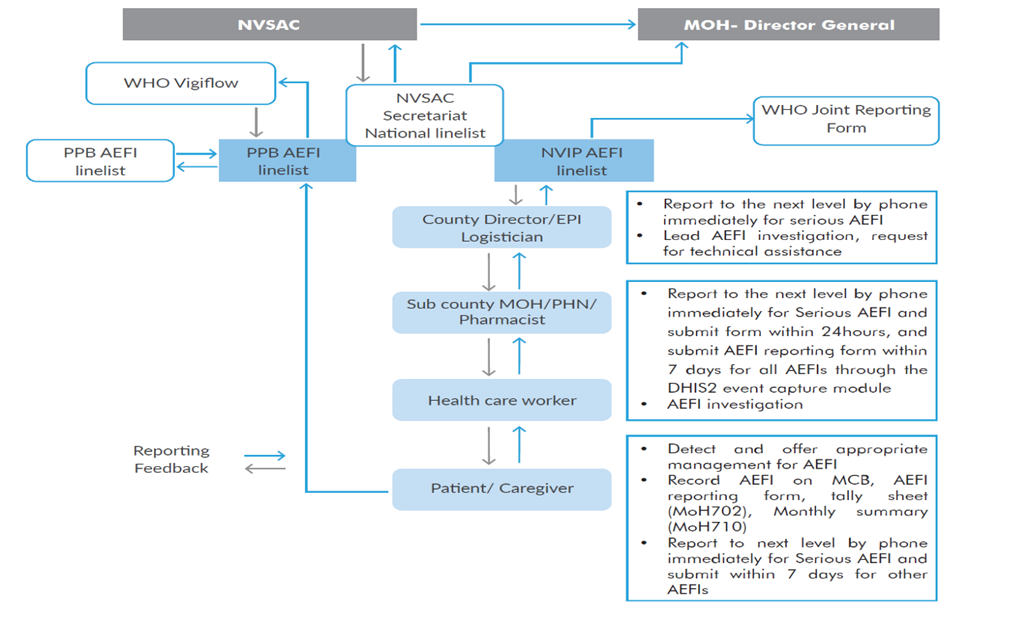


Figure S2: AEFI reporting organogram in Kenya


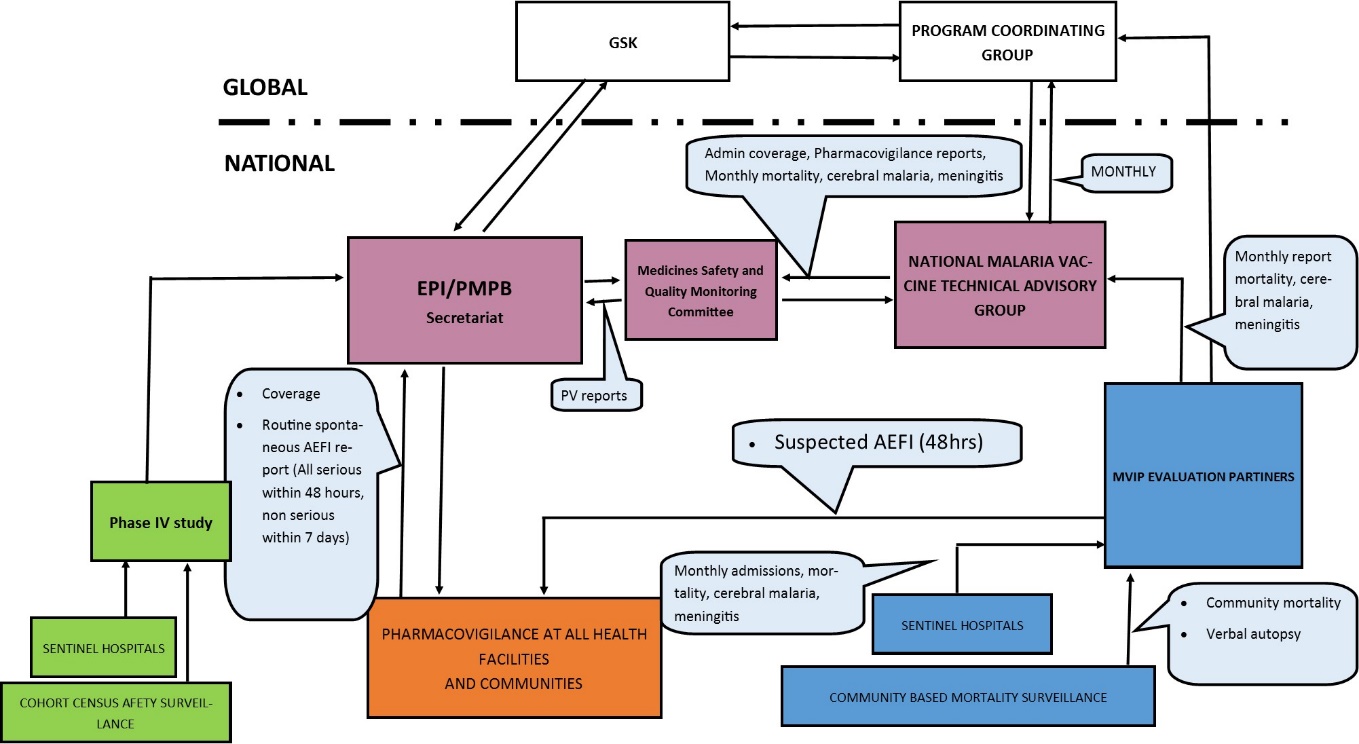


Figure S3: AEFI reporting organogram in Malawi
